# Nerve Injury Following Regional Nerve Block: A Literature Review of Its Etiologies, Risk Factors, and Prevention

**DOI:** 10.1007/s11916-024-01268-w

**Published:** 2024-05-28

**Authors:** Kimmy Bais, Fady Guirguis, Mina Guirguis

**Affiliations:** 1 Department of Anesthesiology, The Ohio State University Wexner Medical Center, Columbus, OH USA; 2 University of Texas at Southwestern Medical Center, Dallas, TX USA

**Keywords:** Peripheral nerve injury, Regional anesthesia, Nerve block, postoperative nerve injury

## Abstract

**Purpose of Review:**

Postoperative nerve injury after nerve block is complex and multifactorial. The mechanisms, etiologies, and risk factors are explored. This review article conducts a literature search and summarizes current evidence and best practices in prevention of nerve injury.

**Recent Findings:**

Emerging technology such as ultrasound, injection pressure monitors, and nerve stimulators for peripheral nerve block have been incorporated into regular practice to reduce the rate of nerve injury. Studies show avoidance of intrafascicular injection, limiting concentrations/volumes of local anesthetic, and appropriate patient selection are the most significant controllable factors in limiting the negative consequences of nerve block.

**Summary:**

Peripheral nerve injury is an uncommon occurrence after nerve block and is obscured by surgical manipulation, positioning, and underlying neural integrity. Underlying neural integrity is not always evident despite an adequate history and physical exam. Surgical stress, independently of nerve block, may exacerbate these neurologic disease processes and make diagnosing a postoperative nerve injury more challenging. Prevention of nerve injury by surgical teams, care with positioning, and avoidance of intrafascicular injection with nerve block are the most evidence-based practices.

## Introduction

### Background

Nerve injury after nerve block is a phenomenon often cited in the regional anesthesia literature by postoperative neurologic symptoms or post-block neurologic dysfunction. This is defined by 2 criteria: patient reported, or evaluator identified sensory, or motor dysfunction present at minimum of 5 days after surgery with an anatomic basis to support the block contributing to neurologic dysfunction [1].

Incidence varies depending on definition of injury: neuraxial injury often combines hematoma, infection, and direct spinal cord injury, all of which depend on the study population, the procedure’s location, and the approach. Multiple studies have led to the America Society of Regional Anesthesia and Pain Medicine to cite the incidence between 1 and 2.2% at 3 months post injury, which falls to 0–0.2% at 1 year [2].

### Anatomy of a Nerve

The peripheral nervous system comprises three types of cells: neuronal, glial, and stromal. Neuronal cells, or peripheral nerves, convey signals between the spinal cord and the body, glial cells such as Schwann cells myelinate nerves, and stromal cells such as endoneurial fibroblasts provide structural support [3]. The structural support comes in the form of connective tissue scaffolding encasing nerves [4]. This scaffolding is composed of three layers: the endoneurium encases individual axons of a nerve, the perineurium circumferentially bundles axons together into fascicles, and the outermost layer, the epineurium is dispersed between fascicles and surrounds the nerve trunk [5].

### Peripheral Nerve Injury

Peripheral nerve injuries (PNIs) are generally graded using the Seddon and Sunderland Classification System for Nerve Injuries [6, 7]. This system grades them on a scale of Grade I to Grade V, with higher grades corresponding to severity. Grade I Injury, known as neurapraxia, involves focal segmental demyelination without any damage to the axons or connective tissue. Grades II through IV, axonotmesis, all involve damage to the axon: in Grade II, the axon is damaged, but the connective tissue is intact, in Grade III, the endoneurium is also damaged, and in Grade IV, the endoneurium and perineurium are damaged in addition to the axon. Finally, a Grade V injury, neurotmesis, is a complete transection of the nerve and connective tissue causing complete discontinuity. The severity of PNI is a general indicator for the recovery a patient can expect following injury: a lower grade injury tends to have near full recovery, while a complete transection has little, if any, return of function following injury without any surgical intervention [8]. 

## Mechanisms of Injury

The American Society of Regional Anesthesia (ASRA) describes four mechanisms of PNI associated with regional anesthesia administration: mechanical, injection, ischemic, and neurotoxic [2]. Mechanical injury, or needle trauma, affecting the perineurium can negate the protective environment of the fascicle, increasing the risk of PNI. Injection of local anesthetic to a denuded axon can cause acute inflammatory reaction or neurotoxicity. Injection can also transiently increase intraneural pressure, leading to ischemia; as can bleeding around the nerve. Finally, other nonspecific inflammatory responses can affect nerves, resulting in neurotoxic damage to the nerve.

### Mechanical Injury

A systematic review by Sondekoppam et al. found that, in animal studies, needle design was a risk factor for PNI following a regional anesthesia block: long-bevel needles were more likely to penetrate fascicular bundles than short-bevel needles, transverse insertion of the bevel is associated with higher amounts of nerve damage than long axis insertion of the nerve, and 17- and 18-G needles caused significantly more fascicular damage than 22-G needles [9]. Another review by Hewson et al. validates this finding and suggest the use of a short-axis view using ultrasound guidance and inserting the needle in-plane to consistently visualize the needle passing through tissue [10]. Mechanical injury can also be caused by mechanical compression from the administration of a tourniquet [11•].

### Ischemic Injury

A review by Hogan discusses ischemic PNI, an injury the severity of which they explain is in proportion to the duration of interruption of blood flow [12]. However, tourniquet-induced neuropathy is only partly caused by ischemia; direct compression of the nerve by the tourniquet also causes nerve damage [12]. Ischemia can cause metabolic stress and parasthesias in the acute phase due to depolarization of sensory neurons; this is then followed by nerve conduction block causing loss of sensation [13]. However, nerve function is not permanently lost at this stage: a study by Lunborg et al. found that nerve function can return within 6 h if ischemic time is less than 2 h [14], and other works have found a return of nerve function after greater than 6 h of ischemia, though the recovery time is much longer [15, 16]. As such, ultrasound plays a key role in administering an effective nerve block with just the minimum amount of local anesthetic at the block site. Hence it avoids ischemic nerve injury due to intraneural injection or use of too much volume of local anesthetic.

### Injection Injury

In addition to direct needle damage, the injection of a needle around or through the nerve may be associated with injuries because of pressure effects. An injection inside the perineurium with a high injection pressure may overcome the compliance of the intraperineural space and cause rupture of the perineurium [17, 18]. This perineurial rupture causes axons to lose the layer protecting them, predisposing to injury [19]. Furthermore, high intrafascicular pressure may result in neural ischemia and inflammation if it exceeds capillary perfusion pressure, exacerbating other types of injuries [20]. A systematic review studying injection pressure for intraneural injections in 2019 validates this finding, reporting that high injection pressures at the needle tip were associated with an increased risk of nerve damage, though no specific cut-off exists [21•].

### Neurotoxic Injury

PNI induced directly by the anesthetic agent is a rare phenomenon with estimated incidence ranging from 4 in 1,000 patients to 3 in one hundred [22, 23]. A review by Nouette-Gaulain et al. reports that the effect local anesthetics have on neurons includes affecting calcium homeostasis, reducing mitochondrial metabolism and thereby increasing oxidative stress, and directly increasing the rate of cell death [24]. The net result of these processes results in a dose-dependent neuronal death, with low doses of anesthetic inducing apoptosis and a high dose inducing necrosis [25–27]. As such, the recommendation by Nouette-Gaulain et al. is to use the minimum local anesthetic concentration needed for the peripheral block and precise targeting of the injection of anesthetic. Another potential solution is injection of a smaller volume with higher concentration, which Nakamura et al. found resulted in greater depth and longer duration of analgesia in an animal model [28]. 

## Etiologies of Nerve Injury

Understanding these mechanisms of injury leads to a further understanding of etiologies of nerve injury which could be related to the patient (preexisting neuropathy), anesthetic (needle trauma), or the surgery (traction injury). Needle injury by the anesthesiologist or surgical manipulation causing transection, stretching, or compression are obvious mechanical causes of nerve injuries. Tourniquet compression, mostly correlated to duration and pressure reached, can lead to postoperative nerve injury [2]. Predisposing neurologic conditions and systemic diseases may decrease the threshold of nerve injury and increase the incidence of postoperative nerve injury. This theory, first introduced as the “**double crush**” hypothesis by Upton and McComas in 1973, states that “neural function is impaired because single axons, having been compressed in one region, become susceptible to damage at another site [29–31]. 

With advancing technology, nerve monitoring techniques have improved exponentially. The simplest, with the patient reporting paresthesias with nerve to needle contact, has not been shown to reduce the chances of postoperative nerve injury [32]. But the absence of paresthesias, in contrast does not exclude possibility of PNI or absence of needle to nerve contact [2, 33]. Patients with neuropathy may not have an appropriate sensory response to nerve stimulation and contact may not elicit paresthesia. Use of nerve stimulator helps confirm appropriate needle placement based on the corresponding motor response by the patient. However, nerve stimulation also may aid in decreasing intraneural injection or needle-nerve injection by using the stimulation threshold of 0.5 mA to gauge needle relationship with nerve (with over 0.5 supporting extraneural or safe placement). Injection pressure monitoring is traditionally done by subjective feel of the syringe. Animal data suggests injection pressure monitoring may be beneficial, but human data has not proven beneficial outcomes [10, 34, 35]. Ultrasound resolution may detect intraneural injection but cannot distinguish intrafascicular vs. interfascicular injection. Ultrasound is also limited by patient anatomy and user expertise to constantly visualize needle tip and nerve relationship. This has led the ASRA expert panels to report evidence statement related to anesthetic risk factors as being primarily surgical or patient related rather than related to peripheral nerve block [2]. True anesthetic consideration would be to avoid intrafascicular injection to reduce neurotoxic axonal injury secondary to local anesthetic exposure [36].

## Addressing Risk Factors for Postoperative Nerve Injury

Patient risk factors predisposing to nerve injury are those which decrease the neural integrity, including systemic metabolic/toxic disease, vascular and neurogenic ischemia. One of the most significant risk factors for postoperative nerve injury is underlying neurologic impairment [37]. One such theory to capture this predisposition, as discussed briefly above, is the **double crush hypothesis**, where proximal neural injuries lead to higher likelihood of distal nerve injury [31, 38, 39]. Double crush was originally described as two sites of physical nerve entrapment with cervical stenosis and carpal tunnel entrapment, with the carpal tunnel syndrome not resolving despite carptal tunnel decompression [31]. In theory, any disruption of axonal signaling may predispose to further injury along the neural tissue [40•, 41]. Neuropathy can be classified as focal (along nerve course) such as nerve entrapment, demyelinating lesions, hereditary neuropathy or diffuse as caused by systemic neuropathy such as ischemic, toxic, metabolic or hereditary etiologies. For example, diabetic neuropathy with microvascular ischemic hypoxia has a higher incidence of PNI than normal neural tissue [33]. Despite the microvascular damage to diabetic nerves, diabetic patients may benefit from regional anesthesia in comparison to general anesthesia given the macrovascular complications of diabetes that predispose to cardiovascular and renal dysfunction that may be exacerbated by general anesthesia. Peripheral vascular disease, smoking, and hypertension may increase ischemic injury and vulnerability to further insults.

Neurologic injury in patients with underlying conditions is challenging to evaluate due to the low prevalence of these conditions and the low incidence of postoperative nerve injury. Moreover, large population studies have difficulty accounting for neurologic impairment that is subclinical or undiagnosed, and the varying severities of neurologic impairment. With the available case series and case reports, Kopp et al. note that hereditary peripheral neuropathy, most commonly Charcot Marie Tooth Disease (CMTD) and hereditary neuropathy with liability to pressure palsy (HNPP) may not be worsened with regional or neuraxial anesthesia, but caution against decreasing surgical or positional risk as well [42, 43••]. Acquired peripheral neuropathies, such as diabetic neuropathy, alcoholic or chemotherapy induced neuropathy, predispose to peripheral nerve injury. While it is unclear exactly how to treat these patients, judicial dosing of local anesthetic and potentially removing epinephrine to decrease ischemic injury to micro-vascularly injured neural tissue may decrease the risk of regional anesthesia [42].

Postsurgical inflammatory neuropathy is a diagnosis where an immune-mediated response to surgical stress response creates aberrant nerve conduction. This pathology may present as focal or diffuse deficits but without anatomic distribution affected by regional anesthesia or surgical procedure. Inflammatory neuropathies such as Guillain-Barre syndrome and postsurgical inflammatory neuropathy have inadequate data to make strong recommendations and use of regional anesthesia should be individualized to patients [2].

For patients with central nervous system disorders, such as multiple sclerosis, post-polio syndrome and amyotrophic lateral sclerosis, patients are likely to exhibit neurologic deficits of various severities. Patients with these illnesses may have stable neurologic deficits and if so may be a candidate for regional anesthesia given the increased likelihood of worsening of neurologic symptoms regardless of anesthetic technique used [2]. A large retrospective study demonstrated no increased exacerbation of multiple sclerosis among obstetric patients [44]. However, anesthesiologists generally err on the side of caution and avoid nerve blocks which would add another risk factor for nerve deficits postoperatively. A recent case report utilized regional anesthesia to avoid airway manipulation in an ALS patient to avoid postoperative ventilation [45•]. This individualized approach for patient and procedure, along with the patient’s priorities and informed consent is likely the best approach for the anesthesiologist to adopt, rather than avoiding nerve blocks in a specific population.

The initial description of the double crush hypothesis entailed spinal canal stenosis as a comorbid injury for patients presenting for carpal tunnel syndrome. Given the remarkable co-incidence of cervical root compression and peripheral nerve entrapment, Upton et al. ascribed the development of these neuropathies to upstream poor axoplasmic flow [31]. Spinal canal stenosis increases with aging which can lead to poor axoplasmic flow from proximal injuries and predispose to distal peripheral injuries. Indeed, a Japanese study found that 64% of patients in their 50s and 93.1% of patients in their 80s may have moderate to severe central stenosis [46]. With this context, spinal canal stenosis or lumbar disk disease may result in new or worsening of neurologic symptoms with regional anesthesia (although this includes neuraxial techniques) [42]. Patients with prior spinal surgery are still candidates for neuraxial or regional anesthesia but it would be prudent to review radiological imaging of their anatomy prior to proceeding.

An additional consideration for peripheral nerve block related injury is anatomical location. Lemke et al. describe increased connective tissue on more distal peripheral nerves relative to proximal nerve which may have higher proportion of sensitive neural architecture [9, 47•]. This connective tissue may help protect from mechanical trauma or chemical neurotoxicity from local anesthetic. However, large studies have refuted the claim that site selection matters with regards to incidence of postoperative nerve injury and the ASRA practice advisory found the site selection based on proximal vs. distal to not be supported by clinical evidence [2].

## Conclusions

Fortunately for the anesthesiologist and patient, the incidence of postoperative nerve injury is rare. Moreover, it is extremely unlikely to be a direct result of nerve block. Postoperative nerve injuries are likely to be neuropraxic and thus short lived with a good prognosis. Patient positioning, surgical mechanics, and patient underlying neural health are far more likely to be significant in increasing the magnitude of peripheral nerve injury. An informed consent process in patients who may have these risk factors is necessary, but the data does not suggest causative associations with regional anesthesia and worsening or new symptoms of neurological injury [2]. While engaging in nerve block, the few things within the operator purview are to minimize possibility of intrafascicular injection or needle placement and to utilize a short bevel needle. This may be done by monitoring of evoked motor response at thresholds below 0.5. While ultrasound may detect intraneural injection, operator and patient factors are unlikely to allow for perfect detection and therefore ultrasound is unlikely to be adequate as a sole identifier for interfascicular vs. intrafascicular injection. Although quantitative injection pressure monitoring is rarely used clinically, intrafascicular injection would be detected by high pressure. Pain or paresthesia are not clearly linked to nerve injury, but for patient comfort should be avoided. While regional block experience is likely to decrease incidence of nerve injury, the incidence is between 1 and 2% at 3 months and decreases to 0.02% at 1 year, so the effects of a nerve injury are unlikely to be permanent. Particularly in patients with preexisting metabolic, neurologic, or vascular conditions that increase the risk of underlying neural compromise, the informed consent process should appropriately counsel these patients regarding their risk of postoperative nerve injury and with the potential benefits of regional anesthesia – alternative to general anesthesia, improved analgesia, and immobility of surgical field – weighed.

## Data Availability

No datasets were generated or analysed during the current study.

## References

[1] Sites BD, Taenzer AH, Herrick MD, Gilloon C, Antonakakis J, Richins J, et al. Incidence of local anesthetic systemic toxicity and postoperative neurologic symptoms associated with 12,668 ultrasound-guided nerve blocks: an analysis from a prospective clinical registry. Reg Anesth Pain Med. 2012;37:478–82. 22705953 10.1097/AAP.0b013e31825cb3d6

[2] Neal JM, Barrington MJ, Brull R, Hadzic A, Hebl JR, Horlocker TT, et al. The second ASRA Practice Advisory on neurologic complications Associated with Regional Anesthesia and Pain Medicine: executive Summary 2015. Reg Anesth Pain Med. 2015;40:401–30. 26288034 10.1097/AAP.0000000000000286

[3] Menorca RMG, Fussell TS, Elfar JC. Nerve physiology: mechanisms of injury and recovery. Hand Clin. 2013;29:317–30. 23895713 10.1016/j.hcl.2013.04.002 PMC4408553

[4] Geuna S, Raimondo S, Ronchi G, Di Scipio F, Tos P, Czaja K, et al. Chapter 3: histology of the peripheral nerve and changes occurring during nerve regeneration. Int Rev Neurobiol. 2009;87:27–46. 19682632 10.1016/S0074-7742(09)87003-7

[5] Rydevik B, Lundborg G. Permeability of intraneural microvessels and perineurium following acute, graded experimental nerve compression. Scand J Plast Reconstr Surg. 1977;11:179–87. 609900 10.3109/02844317709025516

[6] Sunderland S. A classification of peripheral nerve injuries producing loss of function. Brain J Neurol. 1951;74:491–516. 10.1093/brain/74.4.491 14895767

[7] Seddon HJ. Three types of nerve injury. Brain. 1943;66:237–88.

[8] Burnett MG, Zager EL. Pathophysiology of peripheral nerve injury: a brief review. Neurosurg Focus. 2004;16:E1. 15174821 10.3171/foc.2004.16.5.2

[9] Sondekoppam RV, Tsui BCH. Factors Associated with risk of neurologic complications after peripheral nerve blocks: a systematic review. Anesth Analg. 2017;124:645–60. 28067709 10.1213/ANE.0000000000001804

[10] Hewson DW, Bedforth NM, Hardman JG. Peripheral nerve injury arising in anaesthesia practice. Anaesthesia. 2018;73(Suppl 1):51–60. 29313904 10.1111/anae.14140

[11] • Chang J, Bhandari L, Messana J, Alkabbaa S, Hamidian Jahromi A, Konofaos P. Management of Tourniquet-related nerve Injury (TRNI): a systematic review. Cureus. 2022;14:e27685. **Describes the mechanism of nerve injury in procedures utilizing tourniquets.** 10.7759/cureus.27685 PMC9440764 36072167

[12] Hogan QH. Pathophysiology of peripheral nerve injury during regional anesthesia. Reg Anesth Pain Med. 2008;33:435–41. 18774512 10.1016/j.rapm.2008.03.002 PMC2885284

[13] Fern R, Harrison PJ. The contribution of ischaemia and deformation to the conduction block generated by compression of the cat sciatic nerve. Exp Physiol. 1994;79:583–92. 7946287 10.1113/expphysiol.1994.sp003791

[14] Lundborg G. Structure and function of the intraneural microvessels as related to trauma, edema formation, and nerve function. J Bone Joint Surg Am. 1975;57:938–48. 171272

[15] Iida H, Schmelzer JD, Schmeichel AM, Wang Y, Low PA. Peripheral nerve ischemia: reperfusion injury and fiber regeneration. Exp Neurol. 2003;184:997–1002. 14769393 10.1016/S0014-4886(03)00385-6

[16] Tountas CP, Bergman RA. Tourniquet ischemia: ultrastructural and histochemical observations of ischemic human muscle and of monkey muscle and nerve. J Hand Surg. 1977;2:31–7. 10.1016/s0363-5023(77)80007-5 402413

[17] Selander D, Brattsand R, Lundborg G, Nordborg C, Olsson Y. Local anesthetics: importance of mode of application, concentration and adrenaline for the appearance of nerve lesions. An experimental study of axonal degeneration and barrier damage after intrafascicular injection or topical application of bupivacaine (Marcain). Acta Anaesthesiol Scand. 1979;23:127–36. 442943 10.1111/j.1399-6576.1979.tb01432.x

[18] Gentili F, Hudson AR, Hunter D, Kline DG. Nerve injection injury with local anesthetic agents: a light and electron microscopic, fluorescent microscopic, and horseradish peroxidase study. Neurosurgery. 1980;6:263–72. 7383289

[19] Gadsden J, Gratenstein K, Hadzic A. Intraneural injection and peripheral nerve injury. Int Anesthesiol Clin. 2010;48:107–15. 20881530 10.1097/AIA.0b013e3181f89b10

[20] Selander D, Sjöstrand J. Longitudinal spread of intraneurally injected local anesthetics. An experimental study of the initial neural distribution following intraneural injections. Acta Anaesthesiol Scand. 1978;22:622–34. 726868 10.1111/j.1399-6576.1978.tb01346.x

[21] • Carassiti M, De Filippis A, Palermo P, Valenti C, Costa F, Massaroni C, et al. Injection pressures measuring for a safe peripheral nerve block. Minerva Anestesiol. 2019;85:1003–13. **Describes a way to prevent PNI.** 10.23736/S0375-9393.19.13518-3 31124620

[22] Brull R, McCartney CJL, Chan VWS, El-Beheiry H. Neurological complications after regional anesthesia: contemporary estimates of risk. Anesth Analg. 2007;104:965–74. 17377115 10.1213/01.ane.0000258740.17193.ec

[23] Barrington MJ, Watts SA, Gledhill SR, Thomas RD, Said SA, Snyder GL, et al. Preliminary results of the Australasian Regional Anaesthesia collaboration: a prospective audit of more than 7000 peripheral nerve and plexus blocks for neurologic and other complications. Reg Anesth Pain Med. 2009;34:534–41. 19916206 10.1097/aap.0b013e3181ae72e8

[24] Nouette-Gaulain K, Capdevila X, Rossignol R. Local anesthetic in-situ toxicity during peripheral nerve blocks: update on mechanisms and prevention. Curr Opin Anaesthesiol. 2012;25:589–95. 22914357 10.1097/ACO.0b013e328357b9e2

[25] Werdehausen R, Fazeli S, Braun S, Hermanns H, Essmann F, Hollmann MW, et al. Apoptosis induction by different local anaesthetics in a neuroblastoma cell line. Br J Anaesth. 2009;103:711–8. 19700777 10.1093/bja/aep236

[26] Werdehausen R, Braun S, Essmann F, Schulze-Osthoff K, Walczak H, Lipfert P, et al. Lidocaine induces apoptosis via the mitochondrial pathway independently of death receptor signaling. Anesthesiology. 2007;107:136–43. 17585225 10.1097/01.anes.0000268389.39436.66

[27] Johnson ME, Uhl CB, Spittler K-H, Wang H, Gores GJ. Mitochondrial injury and caspase activation by the local anesthetic lidocaine. Anesthesiology. 2004;101:1184–94. 15505455 10.1097/00000542-200411000-00019

[28] Nakamura T, Popitz-Bergez F, Birknes J, Strichartz GR. The critical role of concentration for lidocaine block of peripheral nerve in vivo: studies of function and drug uptake in the rat. Anesthesiology. 2003;99:1189–97. 14576558 10.1097/00000542-200311000-00028

[29] Hebl JR, Horlocker TT, Schroeder DR. Neuraxial anesthesia and analgesia in patients with preexisting central nervous system disorders. Anesth Analg. 2006;103:223–8. table of contents. 16790657 10.1213/01.ane.0000220896.56427.53

[30] Hebl JR, Kopp SL, Schroeder DR, Horlocker TT. Neurologic complications after neuraxial anesthesia or analgesia in patients with preexisting peripheral sensorimotor neuropathy or diabetic polyneuropathy. Anesth Analg. 2006;103:1294–9. 17056972 10.1213/01.ane.0000243384.75713.df

[31] Upton ARM, Mccomas A. The double crush in nerve-entrapment syndromes. Lancet. 1973;302:359–62. 10.1016/s0140-6736(73)93196-6 4124532

[32] Liguori GA, Zayas VM, YaDeau JT, Kahn RL, Paroli L, Buschiazzo V, et al. Nerve localization techniques for interscalene brachial plexus blockade: a prospective, randomized comparison of mechanical paresthesia versus electrical stimulation. Anesth Analg. 2006;103:761–7. 16931693 10.1213/01.ane.0000229705.45270.0f

[33] Lirk P, Birmingham B, Hogan Q. Regional Anesthesia in patients with Preexisting Neuropathy. Int Anesthesiol Clin. 2011;49:144–65. 21956084 10.1097/AIA.0b013e3182101134

[34] Kapur E, Vuckovic I, Dilberovic F, Zaciragic A, Cosovic E, Divanovic K-A, et al. Neurologic and histologic outcome after intraneural injections of lidocaine in canine sciatic nerves. Acta Anaesthesiol Scand. 2007;51:101–7. 17081151 10.1111/j.1399-6576.2006.01169.x

[35] Claudio R, Hadzic A, Shih H, Vloka JD, Castro J, Koscielniak-Nielsen Z, et al. Injection pressures by anesthesiologists during simulated peripheral nerve block. Reg Anesth Pain Med. 2004;29:201–5. 15138903 10.1016/j.rapm.2003.12.013

[36] Farber SJ, Saheb-Al-Zamani M, Zieske L, Laurido-Soto O, Bery A, Hunter D, et al. Peripheral nerve injury after local anesthetic injection. Anesth Analg. 2013;117:731–9. 23921658 10.1213/ANE.0b013e3182a00767

[37] Brull R, Hadzic A, Reina MA, Barrington MJ. Pathophysiology and etiology of nerve Injury following peripheral nerve blockade. Reg Anesth Pain Med. 2015;40:479–90. 25974275 10.1097/AAP.0000000000000125

[38] Hebl JR, Horlocker TT, Pritchard DJ. Diffuse brachial plexopathy after interscalene blockade in a patient receiving cisplatin chemotherapy: the pharmacologic double crush syndrome. Anesth Analg. 2001;92:249–51. 11133638 10.1097/00000539-200101000-00049

[39] Osterman AL. The double crush syndrome. Orthop Clin North Am. 1988;19:147–55. 3275922

[40] • Phan A, Shah S, Hammert W, Mesfin A. Double crush syndrome of the Upper Extremity. JBJS Rev. 2021;9. **Describes the mechanism of nerve injury in patients with underlying neurologic disorderes.** 10.2106/JBJS.RVW.21.00082 34910699

[41] Kane PM, Daniels AH, Akelman E. Double crush Syndrome. J Am Acad Orthop Surg. 2015;23:558–62. 26306807 10.5435/JAAOS-D-14-00176

[42] Kopp SL, Jacob AK, Hebl JR. Regional Anesthesia in patients with preexisting neurologic disease. Reg Anesth Pain Med. 2015;40:467–78. 26115188 10.1097/AAP.0000000000000179

[43] •• McClain RL, Rubin DI, Bais KS, Navarro AM, Robards CB, Porter SB. Regional anesthesia in patients with Charcot-Marie-tooth disease: a historical cohort study of 53 patients. Can J Anaesth J Can Anesth. 2022;69:880–4. **Describes a case series that suggests patient with hereditary neurologic disorder are not at increased risk from regional anesthesia.** 10.1007/s12630-022-02258-5 35469042

[44] Harazim H, Štourač P, Janků P, Zelinková H, Frank K, Dufek M, et al. Obstetric anesthesia/analgesia does not affect disease course in multiple sclerosis: 10-year retrospective cohort study. Brain Behav. 2018;8:e01082. 30047260 10.1002/brb3.1082 PMC6160638

[45] • Samworth AG, Miller K, Haswah M, Tureanu L, Weeks J. Neuraxial and regional anesthesia in a patient with amyotrophic lateral sclerosis: a case report. Cureus [Internet]. 2023 [cited 2024 Apr 10]. https://www.cureus.com/articles/143368-neuraxial-and-regional-anesthesia-in-a-patient-with-amyotrophic-lateral-sclerosis-a-case-report. **Describes a case report where an ALS patient underwent regional anesthesia without complications.** 10.7759/cureus.37364 PMC10170567 37182071

[46] Ishimoto Y, Yoshimura N, Muraki S, Yamada H, Nagata K, Hashizume H, et al. Associations between radiographic lumbar spinal stenosis and clinical symptoms in the general population: the Wakayama Spine Study. Osteoarthritis Cartilage. 2013;21:783–8. 23473979 10.1016/j.joca.2013.02.656

[47] • Lemke E, Johnston DF, Behrens MB, Seering MS, McConnell BM, Swaran Singh TS, et al. Neurological injury following peripheral nerve blocks: a narrative review of estimates of risks and the influence of ultrasound guidance. Reg Anesth Pain Med. 2024;49:122–32. **Provides risk estimate of PNI when using ultrasound.** 10.1136/rapm-2023-104855 37940348

